# *Bacillus coagulans* MTCC 5856 for the management of major depression with irritable bowel syndrome: a randomised, double-blind, placebo controlled, multi-centre, pilot clinical study

**DOI:** 10.29219/fnr.v62.1218

**Published:** 2018-07-04

**Authors:** Muhammed Majeed, Kalyanam Nagabhushanam, Sivakumar Arumugam, Shaheen Majeed, Furqan Ali

**Affiliations:** 1 Sami Labs Limited, Bangalore, Karnataka, India; 2 Sabinsa Corporation, East Windsor, NJ, USA; 3 Sabinsa Corporation, Payson, UT, USA; 4 ClinWorld Private Limited, Bangalore, Karnataka, India

**Keywords:** probiotic, B. coagulans MTCC 5856, LactoSpore^®^, major depression, irritable bowel syndrome

## Abstract

**Background:**

The modification of microbial ecology in human gut by supplementing probiotics may be an alternative strategy to ameliorate or prevent depression.

**Objective:**

The current study was conducted to assess the safety and efficacy of the probiotic strain *Bacillus coagulans* MTCC 5856 for major depressive disorder (MDD) in IBS patients.

**Method:**

Patients (*n* = 40) diagnosed for MDD with IBS were randomized (1:1) to receive placebo or *B. coagulans* MTCC 5856 at a daily dose of 2 × 109 cfu (2 billion spores) and were maintained to the end of double-blind treatment (90 days). Changes from baseline in clinical symptoms of MDD and IBS were evaluated through questionnaires.

**Results:**

Significant change (*p* = 0.01) in favour of the *B. coagulans* MTCC 5856 was observed for the primary efficacy measure Hamilton Rating Scale for Depression (HAM-D), Montgomery-Asberg Depression Rating Scale (MADRS), Center for Epidemiological Studies Depression Scale (CES-D) and Irritable bowel syndrome quality of life questionnaire (IBS-QOL). Secondary efficacy measures i.e. Clinical Global Impression-Improvement rating Scale (CGI-I), Clinical Global Impression Severity rating Scale (CGI-S), Gastrointestinal Discomfort Questionnaire (GI-DQ) and Modified Epworth Sleepiness Scale (mESS) also showed significant results (*p* = 0.01) in *B. coagulans* MTCC 5856 group compared to placebo group except dementia total reaction scoring. Serum myeloperoxidase, an inflammatory biomarker was also significantly reduced (*p* < 0.01) when compared with the baseline and end of the study. All the safety parameters remained well within the normal clinical range and had no clinically significant difference between the screening and at the end of the study.

**Conclusion:**

*B. coagulans* MTCC 5856 showed robust efficacy for the treatment of patients experiencing IBS symptoms with major depressive disorder. The improvement in depression and IBS symptoms was statistically significant and clinically meaningful. These findings support *B. coagulans* MTCC 5856 as an important new treatment option for major depressive disorder in IBS patients.

Major depressive disorder (MDD) is characterised by an increased medical morbidity, mortality, feelings of guilt, low mood, reduced quality of life, disturbed sleep or appetite (1). MDD is one of the most common mental disorders worldwide, with a life time prevalence of 16.2% and a 12-month prevalence of 6.6% in developed countries (2, 3). Furthermore, between 30 and 40% of patients who suffer from MDD never achieve symptom resolution with standard antidepressant treatment (4). Alternative approaches such as cognitive behavioural therapy and lifestyle interventions need highly trained therapists and several weeks to months to achieve effectiveness (5). Therefore, there is a need for new and additional treatment options for depression.

Irritable bowel syndrome (IBS) is characterised by the alterations in bowel function or discomfort, abdominal pain or bloating, and diarrhoea or constipation (6). The prevalence of IBS is estimated between 9 and 23% in the population across the world (6–8) and affects ~21% of the population in South America and ~7% of the population in Southeast Asia (9). Although IBS is classified as a functional gastrointestinal disorder which is a chronic condition, recent developments suggested that IBS may have an impact on extra-intestinal symptoms including genitourinary, musculoskeletal, headaches and fatigue, menstrual, sexual dysfunction, anxiety and mood disorders (7–9). Additionally, clinical symptoms of IBS have been linked with quality of sleep and had co-relation with dementia (10). Thus, patients diagnosed with IBS require specific attention to all psychosocial factors involved including major depression. It has been proposed that gut microbiota is a complex community of over 100 trillion microbial cells, outnumbering the human cells in human bodies by a factor of 10 (11). However, recent findings by Ron Sender et al. (12) suggested that the ratio of bacterial to host cells in humans could be closer to 1:1 which is much lesser compared to earlier reports (12). Regardless of the number of gut microbes reported by various researchers, the role of gut microbiota has been reported to influence human physiology, metabolism, nutrition and immune function, while disruption of gut microbiota has been linked with GI conditions such as IBD, obesity and metabolic disorder (13). It has been postulated that the communication between the gut and brain has multiple routes which include the vagus nerve (VN), the immune system, short-chain fatty acids and tryptophan. However, recent research further suggests that the communication between the gut and the brain may be influenced by the number of the factors, that is, integrity of the intestinal wall, composition/diversity of the gut microbiota and also production of neurotransmitters, hormones, and immune- and neuropeptides by the microbiota in gut (14). Further, several studies have revealed the differences in the composition of the gut microbiota between IBS patients and healthy controls (15). The association between MDD and altered gut microbiota may result in carbohydrate malabsorption and could be associated with mental depression which is defined as a common mental disorder that causes people to experience depressed mood, loss of interest or pleasure, feelings of guilt or low self-worth, disturbed sleep or appetite, low energy and poor concentration (15, 16). Gut microbiota may also be implicated in brain autoimmunity in multiple sclerosis (17). Probiotics are live microorganisms which, when administered in adequate amounts, confer a health benefit on the host (18). Gut microbiota produce immune activating and other signalling molecules (e.g. tryptophan) that may play an important role in regulating the brain and subsequent behaviour (15). On the contrary, bacterial products, such as the Gram-negative endotoxins, can influence mood and cognitive functions (19). Therefore, the modification of microbial ecology by supplementing probiotics may be an alternative option for the management of anxiety and depression (15, 20). LactoSpore^®^, a commercial proprietary probiotic preparation, is lactose free, non-GMO with GRAS status which contains spores of *B. coagulans* MTCC 5856 strain (bearing the internal reference number SBC37-01) (21). There are several commercial preparations of *B. coagulans* reported for the treatment of vaginal infections, adjuvant to antibiotic therapy and lactose intolerance, in human clinical trials (22). *Bacillus coagulans* MTCC 5856 can withstand high temperature and is found to be stable during processing and storage conditions of functional foods (baked foods, brewed coffee, etc.) (23). *Bacillus coagulans* MTCC 5856 elicited anti-diarrhoeal activity and inhibited the gastrointestinal motility in an animal model (24). *Bacillus coagulans* MTCC 5856 was found to be safe and tolerable at a dose of 2 × 10^9^ cfu (spores)/day in humans (25). Further, *B. coagulans* MTCC 5856 at a dose of 2 × 10^9^ cfu (spores)/day along with standard care of treatment was found to be safe and effective in diarrhoea-predominant IBS patients (22). The effect of *B. coagulans* MTCC 5856 has not yet been evaluated in patients experiencing IBS symptoms with MDD. Thus, a 90-day randomised, double-blind, placebo-controlled, multi-centre clinical trial was designed to evaluate the safety and efficacy of *B. coagulans* MTCC 5856 at a dose of 2 × 10^9^ cfu (spores)/day in patients experiencing IBS symptoms with MDD.

## Study rationale

The relationships between psychological distress and gastrointestinal symptoms have been reported earlier (26), and most of the patients suffering from IBS identify stress and anxiety as symptom aggravators (27). Microbes are the important link for the communication of the gut–brain axis, and the alteration of gut–brain axis by traditional medicines will be a potential strategy for the management of comorbid central nervous system (CNS) disorders and gastrointestinal problems (15). This suggests that the probiotics or gut microbes may be a useful therapeutic option in stress-related disorders such as depression and anxiety (13, 20). Recent research suggests that the beneficial gut microorganisms may improve the host health both physically and mentally and may be involved in the development of the neural system and behavioural pattern (28). Thus, this placebo-controlled clinical trial was carried out at three sites using *B. coagulans* MTCC 5856 probiotic in patients with IBS and depression to test the hypothesis that *B. coagulans* can help in alleviating the symptoms of major depression in patients with IBS.

## Experimental procedures

### Product description

The investigational product in this study was *B. coagulans* MTCC 5856 tablets (600 mg) that contained 2 billion spores (333.33 mg), microcrystalline cellulose, starch, sodium starch glycolate and magnesium stearate. Placebo tablets had same ingredients except *B. coagulans* MTCC 5856. Placebo and investigational products were identical in terms of packaging, taste, colour and texture. *B. coagulans* MTCC 5856 spore count in the tablet was determined by following pour plate method as described previously (21).

### Ethics and informed consent

This trial was conducted in accordance with the clinical research guidelines established by the Drugs and Cosmetics Act, 1940 of India; Drugs and Cosmetics Rules, 1945 of India; Ethical Guidelines for Biomedical Research on Human Participants, 2006 of Indian Council of Medical Research (ICMR) in India; the principles enunciated in the Declaration of Helsinki, Edinburgh (29) (Anonymous, 2000); and the International Conference on Harmonisation (ICH) – harmonised tripartite guideline regarding Good Clinical Practice (GCP). The study was approved by the local ethics committee, a written informed consent was obtained and the study was registered at Clinical Trials Registry − India (www.ctri.nic.in) (identifier: CTRI/2015/05/005754 on 06 May 2015). There were no changes to the methods or planned endpoints after study initiation.

### Participants

Subjects were included in the study if indicated ‘Yes’ to all of the inclusion criteria and ‘No’ to all of the exclusion criteria.

### Inclusion criteria

1. Male and/or female subjects ranging in age between 20 and 65 years.
2. Fulfilling Rome III Diagnostic Criteria (30) for Functional IBS. Criterion fulfilled for the last 3 months with symptom onset at least 6 months prior to diagnosis:
  - Discomfort or recurrent abdominal pain at least 3 days/month in the last 3 months associated with two or more of the following: improvement with defecation, stool frequency change and change in appearance of stool
  - Bloating or visible distension at least 3 days/month in the last 3 months
  - Watery or loose stools without pain occurring in at least 75% of stools
3. Willingness to follow the protocol requirement as evidenced by written informed consent.
4. Diagnosed patients with mild to moderate IBS in severity with possible sleep, pain and dementia-associated co-morbidities.
5. Fulfilling Diagnostic and Statistical Manual of Mental Disorders, 4th Edition (2000) Criteria for MDD.
6. Willingness to complete subject diaries and study questionnaires.
7. Agree not to use any medication (prescription and over the counter), including vitamins and minerals, during the course of this study.
8. Agree not to use any yogurt during the course of this study.
9. Subjects whose blood chemistries are within a normal range or not considered clinically significant if outside the normal range.
10. Subject’s assurance that they have not taken antibiotics or other supplements whose primary site of action is in the gastrointestinal tract for a period up to 1 month prior to the start of the study.
11. Willing to come for regular follow-up visit.

### Exclusion Criteria

1. Any clinically significant medical history, medical finding or an ongoing medical condition exists which in the opinion of the investigator could jeopardise the safety of the subject, impact validity of the study results or interfere with the completion of study according to the protocol.
2. Significant abnormal findings as determined by baseline history, physical examination, vital signs, haematology, serum chemistry and urinalysis.
3. History or presence of significant alcoholism or supplement/drug abuse in the past 1 year.
4. Any medical or surgical conditions which might significantly interfere with the gastrointestinal tract, liver, kidneys and/or blood-forming organs.
5. History of cardiovascular, renal, hepatic, asthma, glaucoma, pulmonary, neurologic, metabolic or psychiatric disease.
6. Participation in a clinical study during the preceding 90 days.
7. History of malignancy or other serious disease.
8. Any contraindication to blood sampling.
9. Smoking or consumption of tobacco products.
10. Blood or blood products donated in past 30 days prior to study supplement administration.
11. Pregnant female subjects and lactating women.
12. Prior surgical therapy for obesity.
13. Patients using yogurt in their daily meal.

## Trial Design

**Fig. 1:**
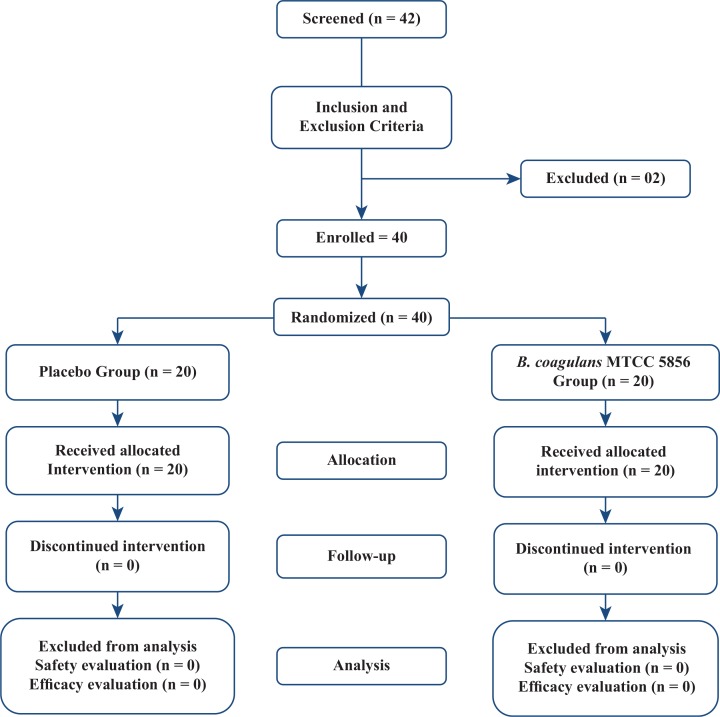
Flow chart of study procedures.

The disposition of the study participants is depicted in Fig. 1. This randomised multi-centre, double-blind, placebo-controlled, parallel-group clinical trial was conducted in India between June 2015 and October 2015 at three different sites: (1) Life Care Hospital, Bangalore, India; (2) Sri Venkateshwara Hospital, Bangalore, India; and (3) Sapthagiri Institute of Medical Sciences and Research Centre, Bangalore, India. The sample size of the study was 40, with 20 subjects randomised to each of the two study arms in a double-blinded manner at a 1:1 ratio. Subjects were blinded and received investigation products dispensed as per randomisation code provided at each site by an authorised person independently. Compliance with study supplement was reviewed at each visit. The daily food intake of the patients was recorded in the patient diaries provided to them at Visit 1 (Day 0). The same was checked and verified at subsequent visits by the investigators. Participants were accompanied by caretakers for their respective visit to the study sites and also were overseen by caretakers during the entire duration of the study. The study consisted of a 90-day intervention period. Subjects visited the study site on screening, baseline/randomisation visit, day 30, day 60, day 90 and day 105. A description of visits 1, 2, 3 and 4 with schedule of events is provided in Table 1.

**Table 1:** Schedule of events

Procedure	Screening	Visit 1 (Day 0) Baseline	Visit 2 (Day 30)	Visit 3 (Day 60)	Visit 4 (Day 90) Final visit	Follow-Up visit (At least 15 days from the last visit)
Informed consent	X					
Medical history	X					
Physical examination	X	X	X	X	X	
Demographics^a^	X	X	X	X	X	
Vital Signs	X	X	X	X	X	
Haematology	X				X	
Serum chemistry	X				X	
Myeloperoxidase^b^		X			X	
Urine pregnancy test^c^	X					
Randomisation		X				
Investigational product dispensing		X	X	X		
Gastrointestinal discomfort questionnaire		X	X	X	X	
Irritable bowel syndrome quality of life questionnaire		X	X	X	X	
HAM-D scale		X	X	X	X	
MADRS		X	X	X	X	
CGI-I & CGI-S		X	X	X	X	
CES-D		X	X	X	X	
Dementia checklist		X	X	X	X	
Return of unused IP			X	X	X	
Adverse events		X	X	X	X	X
Concomitant medications	X	X	X	X	X	X

HAM-D, Hamilton Rating Scale for Depression; MADRS, Montgomery–Asberg Depression Rating Scale; CGI-I & CGI-S, Clinical Global Impression-Improvement Rating Scale and Clinical Global Impression Severity Rating Scale; CES-D, Center for Epidemiological Studies Depression Scale.

a Age at screening only.

b Only for randomised subjects.

c Urine pregnancy test at screening and on early termination, if any.

## Intervention

Newly diagnosed patients and patients who were not on any other treatment for major depression with IBS in the past 3 months were enrolled into the study. This criterion was based on physical, medical and medication history of subjects. All enrolled subjects were instructed to self-administer one tablet per day (either *Bacillus coagulans* MTCC 5856 or placebo) at least 30 min before meal, in the morning as dietary supplement for a period of 90 days.

## Efficacy Outcomes

The primary outcome for this study was a mean 90-day change in depression and IBS symptoms as assessed by the Hamilton Rating Scale for Depression (HAM-D) (31), Montgomery–Asberg Depression Rating Scale (MADRS) (32, 33), sleep quality and depressive symptom severity using 11-item Centre for Epidemiological Studies–Depression Scale (CES-D) (34) and Irritable Bowel Syndrome Quality of Life (IBS-QOL) questionnaire (35). Additionally, secondary efficacy assessments included change in Clinical Global Impression-Improvement (CGI-I) Rating Scale, Clinical Global Impression Severity (CGI-S) Rating Scale (36), Dementia – Revised Memory and Behaviour Problem Checklist (RMBPC) (37), Gastrointestinal Discomfort Questionnaire (GI-DQ) (38) and Modified Epworth Sleepiness Scale (mESS) (39) from the baseline till the end of study as subjective test.

## Bioassays

Serum myeloperoxidase, an inflammatory biomarker, was also analysed for both the groups (*B. coagulans* MTCC 5856 and placebo) at baseline and end of the study. The serum myeloperoxidase levels were measured by implementation of enzyme-linked immunosorbent assay (ELISA) techniques according to manufacturer directions (Cayman Chemicals Company, MI, USA).

## Safety outcomes

Safety of the study was assessed considering the occurrence of adverse events (AEs), safety blood parameters and change in vital signs (blood pressure and heart rate). Laboratory data were summarised by presenting summary statistics of raw data and change in laboratory values from baseline to end of study relative to normal reference limits. Descriptive physical examinations such as abdomen, extremities, general appearance, head, ear, nose, throat, heart, lungs and neurological were monitored at screening, day 0, day 30, day 60, day 90 and day 120 for safety evaluation. Spontaneously reported or observed AE was assessed at all post-screening study visits. AE was evaluated in terms of intensity (mild, moderate or severe) and possible relationship to the study product. There was only one AE reported which was fever and weakness, which was unrelated and not attributable to the study product because the subject who reported this AE belonged to the placebo group.

## Statistical analysis

Statistical Analysis Software (SAS) version 9.2 software was used for data analysis here. Paired ‘*t*’ test, Analysis of Covariance (ANCOVA) and Wilcoxon signed-rank sum test were used for appropriate data set variables to reach the best possible statistical conclusion between the *B. coagulans* MTCC 5856-receiving and placebo-receiving groups. Last Observation Carry Forward (LOCF) method was followed for efficacy evaluations of subjects. No formal sample size calculation was performed.

## Results

### Patient disposition and characteristics

A total of 42 subjects were screened and 40 were enrolled into the study. There were no patient withdrawals or dropouts in this study. Treatment compliance across various visits was checked by the study personnel, and overall treatment compliance for the whole study revealed that 24 (60%) patients met 100% treatment compliance on visit 4 (end of the study) and on an average 66.7% of participants met with 100% treatment compliance during the whole study period (across various visits). At baseline visit (Day 0), no significant difference was observed between the two treatment groups in subject demographics (Table 2). None of the enrolled subjects had abnormal medical history, except for gastrointestinal. Around 3 subjects (7.50%) had earlier GI-related medical history which had no interference with IBS.

**Table 2:** Demographics and baseline clinical characteristics

	Placebo (*n* = 20)	*Bacillus coagulans* MTCC 5856 (*n* = 20)
Sex, *n* (%)		
Female	17 (85)	17 (85)
Male	03 (15)	03 (15)
Age (years), mean (SD)		
	43.88 ± 9.85	40.36 ± 10.28
Height (cm), mean (SD)		
	157.39 ± 8.49	160.1 ± 7.87
Body mass index (kg/m2)		
	25.9 ± 4.49	25.4 ± 4.46
Smokers, *n* (%)		
Ex-smoker	18 (90)	19 (95)
Non-smoker	01 (5)	00
Smoker	01 (5)	01 (5)
Race, *n* (%)		
Central American	00	00
East Asian	00	00
South Asian	20 (100)	20 (100)
South American	00	00
South East Asian	00	00
Western European	00	00
White	00	00
Alcohol use		
Non-drinker	01 (5)	00
Past drinker	18 (90)	19 (95)
Occasional drinker	01 (5)	01 (5)
Current drinker	00	00
Baseline data, mean (SD)		
IBS-QOL	102.6 ± 21.11	106.4 ± 23.44
CGI-I	3.8 ± 1.01	3.7 ± 0.87
CGI-S	3.7 ± 0.92	3.4 ± 0.96
HAM-D	14.5 ± 3.41	13.6 ± 4.41
MADRS	17.1 ± 4.63	16.3 ± 5.40
CES-D	20.7 ± 4.86	19.1 ± 5.25
Dementia – total frequency scoring	61.3 ± 19.11	62.3 ± 17.08
Dementia – total reaction scoring	61.0 ± 19.83	63.8 ± 17.57
mESS	10.9 ± 2.99	10.3 ± 2.43
GI-DQ	32.5 ± 13.88	30.1 ± 15.07

CES-D, Center for Epidemiological Studies Depression Scale; CGI-I, Clinical Global Impression-Improvement Rating Scale; CGI-S, Clinical Global Impression Severity Rating Scale; CI, confidence interval; GI-DQ, Gastrointestinal Discomfort Questionnaire; HAM-D, Hamilton Rating Scale for Depression; IBS-QOL, Irritable Bowel Syndrome Quality of Life Questionnaire; MADRS, Montgomery–Asberg Depression Rating Scale; mESS, Modified Epworth Sleepiness Scale.

Values are expressed as mean ± S.D.

### Efficacy evaluation

**Fig. 2:**
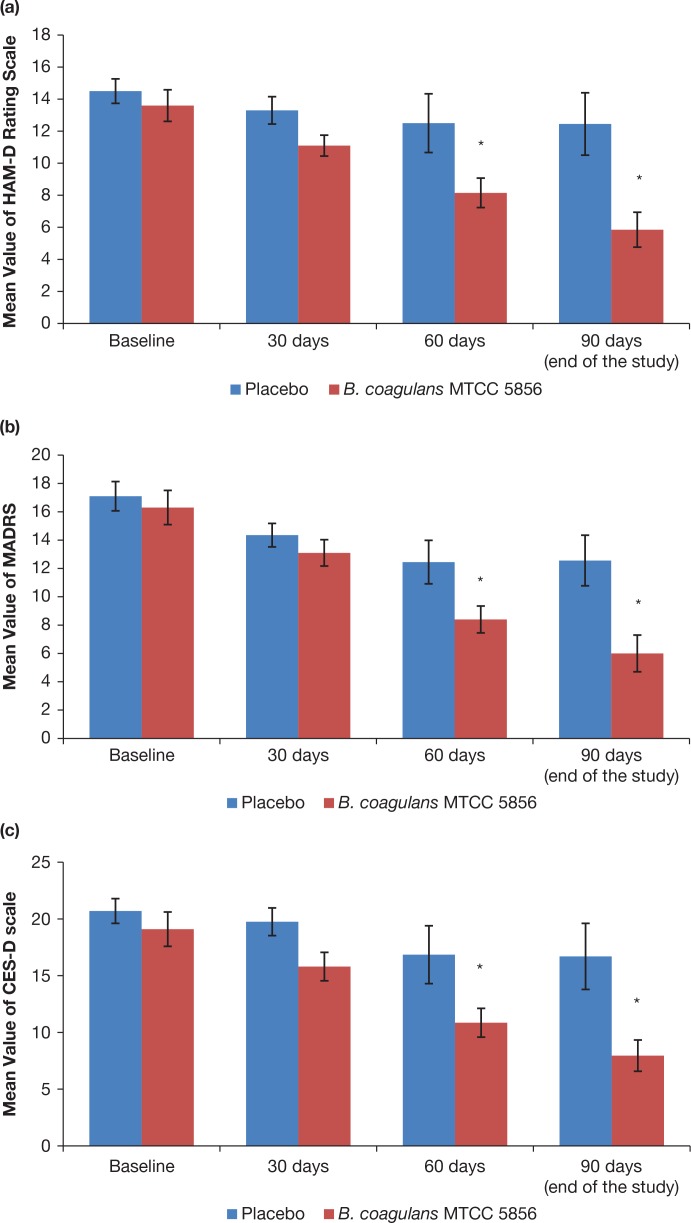

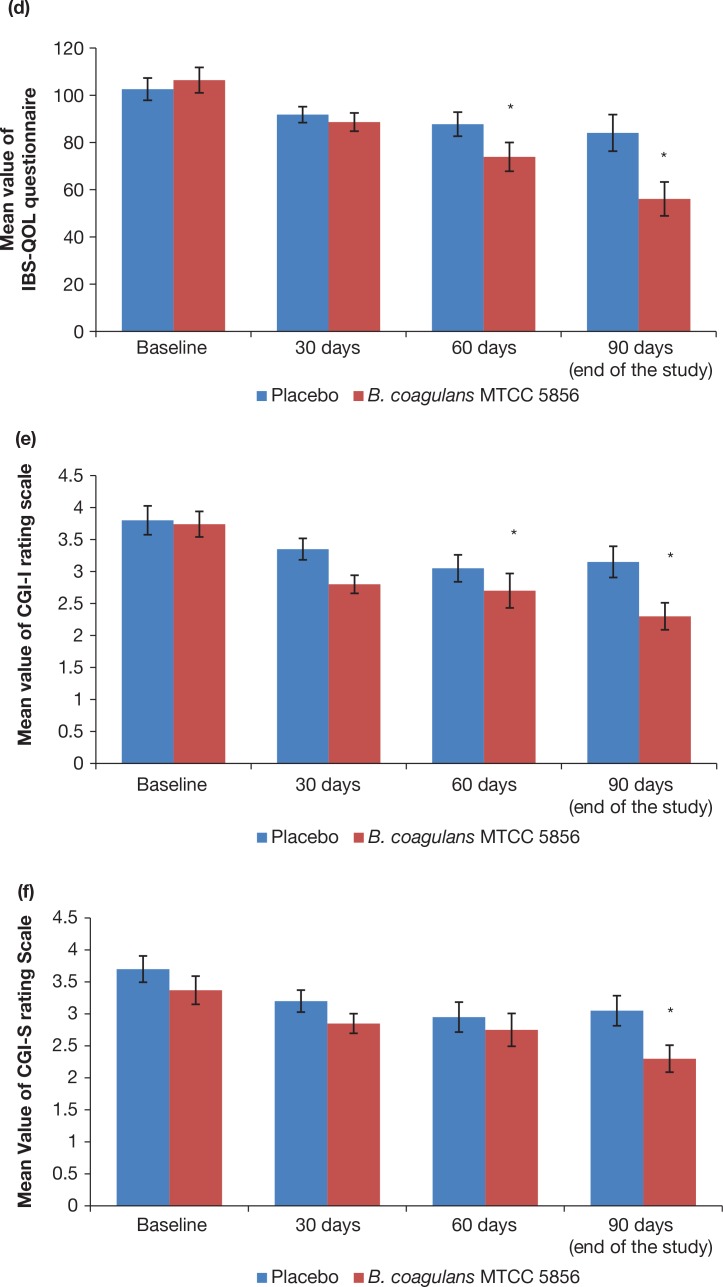

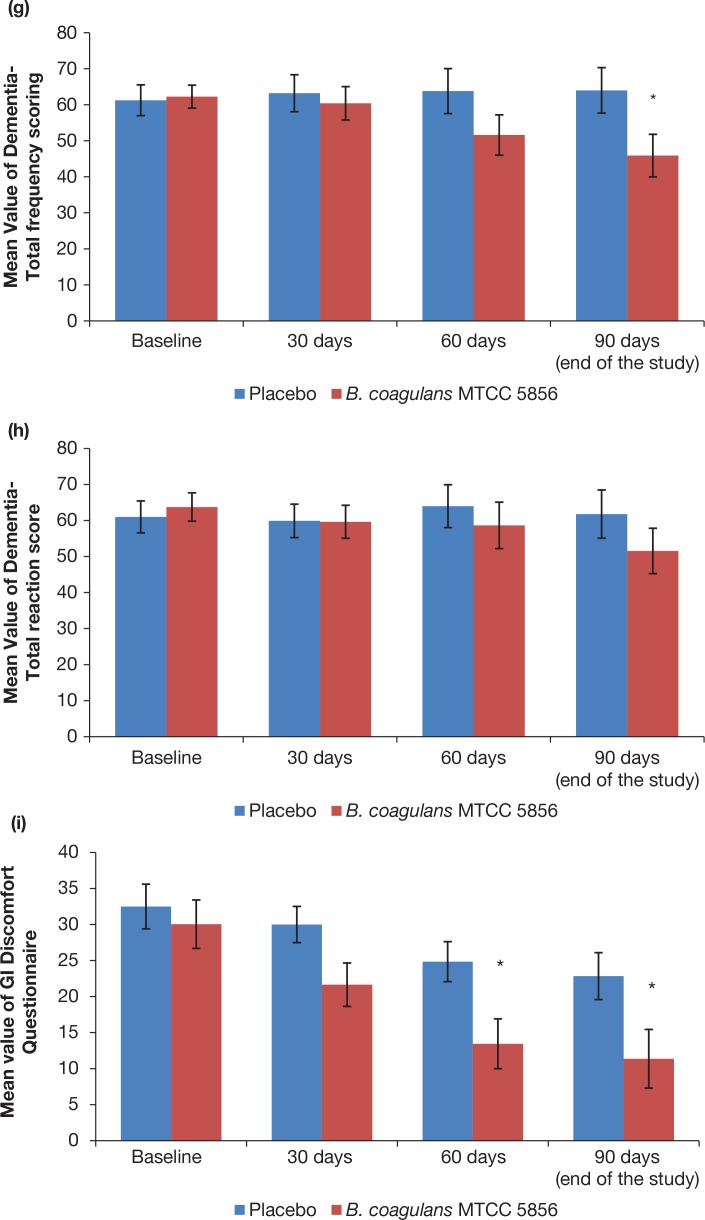

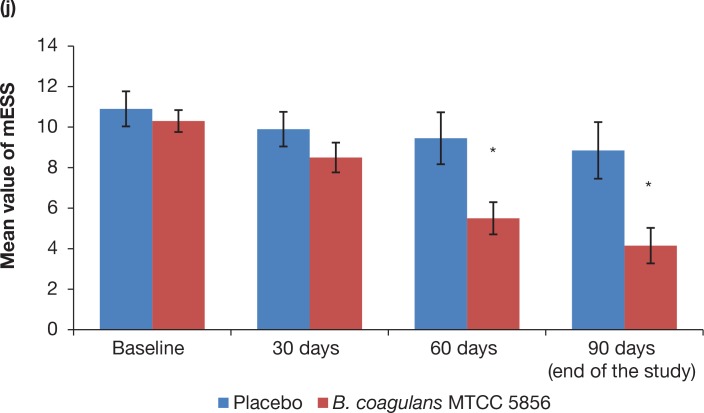
Primary and secondary efficacy measures at baseline, 30, 60 and 90 days (end of the study). All the values are expressed as mean ± S.E. (a) Hamilton Depression Rating Scale (HAM-D) on a scale of 0 to 20. (b) Montgomery–Asberg Depression on a scale of 0 to 20. (c) Center for Epidemiologic Studies Depression on a scale of 0 to 30. (d) IBS – Quality of Life score on a scale of 150. High QOL value indicates poor quality of life. (e) Clinical Global Impression-Improvement Rating on a scale of 0 to 5. (f) Clinical Global Impression-Severity Rating on a scale of 0 to 5. (g) Dementia – Total Frequency Scoring on a scale of 0 to 100. (h) Dementia – Total Reaction Scoring on a scale of 0 to 100. (i) Gastrointestinal Discomfort Questionnaire on a scale of 0 to 40. Low value indicates less GI discomfortness. (j) Modified Epworth Sleepiness scoring on a scale of 0 to 15. *p < 0.05 between the treatment groups and also between baseline and end of the study (day 90).

Statistical analysis using Analysis of Co-Variance (ANCOVA) showed that the primary efficacy parameters were found to be statistically significant (*p* < 0.01) between the *B. coagulans* MTCC 5856 and placebo groups (Table 3). Similarly, all secondary efficacy parameters were also found to be statistically significant between the *B. coagulans* MTCC 5856 and placebo groups except for ‘Dementia total reaction scoring’ (Table 3). Furthermore, comparative mean values of efficacy assessments between *B. coagulans* MTCC 5856 and placebo groups across various visits (baseline, day 30, day 60 and day 90) are as presented for primary efficacy parameters (Fig. 2a–d) and secondary efficacy parameters (Fig. 2e–j). Patients who were on *B. coagulans* MTCC 5856 had statistically significant difference for the efficacy parameters on day 60 and maintained till the end of the study when compared with placebo (Fig. 2).

**Table 3:** Summary of efficacy outcomes at the end of study (day 90): (full analysis set, last observation carried forward, ANCOVA model, 95% CI)

Efficacy parameters	*Bacillus coagulans* MTCC 5856 (*n* = 20)	Placebo (*n* = 20)	Δ *p*-value
**Primary efficacy outcomes**			
HAM-D			
Base line (Day 0)	13.6 ± 4.41	14.5 ± 3.41	0.474
End of the study (Day 90)	5.9 ± 4.88	12.5 ± 8.70	0.005*
Change from baseline to day 90	*p* ≤ 0.001*	*p* = 0.333	0.029*
MADRS			
Base line (Day 0)	16.3 ± 5.40	17.1 ± 4.63	0.618
End of the study (Day 90)	6.0 ± 5.79	12.6 ± 8.00	0.007*
Change from baseline to day 90	*p* ≤ 0.001*	*p* = 0.056	0.031*
CES-D			
Base line (Day 0)	19.1 ± 5.25	20.7 ± 4.86	0.323
End of the study (Day 90)	8.0 ± 6.17	16.7 ± 13.03	0.009*
Change from baseline to day 90	*p* ≤ 0.001*	*p* = 0.224	0.051*
IBS-QOL			
Base line (Day 0)	106.4 ± 23.44	102.6 ± 21.11	0.595
End of the study (Day 90)	56.1 ± 31.26	84.1 ± 34.67	0.010*
Change from baseline to day 90	*p* ≤ 0.001*	*p* = 0.075	0.027*
**Secondary efficacy outcomes**			
CGI-I			
Base line (Day 0)	3.7 ± 0.87	3.8 ± 1.01	0.835
End of the study (Day 90)	2.3 ± 0.92	3.2 ± 1.09	0.011*
Change from baseline to day 90	*p* ≤ 0.001*	*p* = 0.096	0.141
CGI-S			
Base line (Day 0)	3.4 ± 0.96	3.7 ± 0.92	0.277
End of the study (Day 90)	2.3 ± 0.92	3.1 ± 1.05	0.022*
Change from baseline to day 90	*p* = 0.009*	*p* = 0.058	0.396
Dementia – TFS			
Base line (Day 0)	62.3 ± 17.08	61.3 ± 19.11	0.862
End of the study (Day 90)	45.9 ± 26.42	64.0 ± 28.26	0.043*
Change from baseline to day 90	*p* = 0.0046*	*p* = 0.592	0.011*
Dementia – TRS			
Base line (Day 0)	63.8 ± 17.57	61.0 ± 19.83	0.645
End of the study (Day 90)	51.6 ± 28.19	61.8 ± 29.94	0.118
Change from baseline to day 90	*p* = 0.047*	*p* = 0.880	0.103
GI-DQ			
Base line (Day 0)	30.1 ± 15.07	32.5 ± 13.88	0.596
End of the study (Day 90)	11.4 ± 18.23	22.9 ± 14.55	0.035*
Change from baseline to day 90	*p* ≤ 0.001*	*p* = 0.058	0.132
mESS			
Base line (Day 0)	10.3 ± 2.43	10.9 ± 2.99	0.490
End of the study (Day 90)	4.2 ± 3.92	8.9 ± 6.24	0.007*
Change from baseline to day 90	*p* ≤ 0.001*	*p* = 0.171	0.018*

**Δ** Between-group comparisons were made using the ANCOVA. Within-group comparisons were made using the paired Student’s *t*-test. *Probability (*p*) values ≤0.05 are statistically significant.

ANCOVA, analysis of covariance; CES-D, Center for Epidemiological Studies Depression Scale; CGI-I, Clinical Global Impression-Improvement rating Scale; CGI-S, Clinical Global Impression Severity Rating Scale; CI, confidence interval; GI-DQ, Gastrointestinal Discomfort Questionnaire; HAM-D, Hamilton Rating Scale for Depression; IBS-QOL, Irritable Bowel Syndrome Quality of Life Questionnaire; MADRS, Montgomery–Asberg Depression Rating Scale; mESS, Modified Epworth Sleepiness Scale; Dementia – TFS, Dementia – Total frequency scoring; Dementia – TRS, Dementia – Total reaction scoring.

* *p*-value significant (<0.05).

Values are expressed as mean ± SD.

## Bioassay

The level of serum myeloperoxidase was significantly reduced (*p* < 0.01) from the baseline to the end of study (day 90) in patients receiving 2 × 10^9^ spores (cfu)/day of *B. coagulans* MTCC 5856 (Table 4). However, no significant change in the level of serum myeloperoxidase was observed in the placebo group (*p* > 0.05) from the baseline to the end of study (day 90) (Table 4).

**Table 4:** Effect of *Bacillus coagulans* MTCC 5856 on the serum myeloperoxidase

Serum myloperoxidase (ng/mL)			

	*Bacillus coagulans* MTCC 5856 (*n* = 20)	Placebo (*n* = 20)	*p*-value
Baseline (Day 0)	12.6 ± 4.31	9.81 ± 4.10	*p* = 0.0460
End of the study (Day 90)	7.7 ± 2.57	9.8 ± 4.65	*p* ≤ 0.01
Change from baseline to day 90	*p* ≤ 0.01	*p* = 0.9886	*p* ≤ 0.01

Serum myeloperoxidase was quantified at baseline (Day 0) and end of the study (day 90) of both *B. coagulans* MTCC 5856 and placebo group. ANCOVA was performed between the group and *t*-test was performed within the group. Values expressed as mean ± S.D. *p* < 0.05 considered as significant.

## Safety Evaluations

Vital signs such as blood pressure, respiratory rate, pulse rate and any abnormal lab/diagnostic parameters were considered for safety evaluations. No clinically significant changes were recorded for descriptive physical examination in either of the group. Further, no clinically significant abnormal lab values (biochemistry and haematology) were identified (Table 5), and no statistically significant changes in the vitals were observed from the baseline to final visit (Table 6). No serious AEs or significant AEs were noticed in this study. There was only one AE reported, fever and weakness, which resolved without the use of any concomitant medication(s). The unblinding of study product code towards end of the study revealed that the patient with AE belonged to the placebo group.

**Table 5:** Biochemistry and haematology values between two treatment groups

Lab parameter (units)	Visit	Placebo (*n* = 20)	*Bacillus coagulans* MTCC 5856 (*n* = 20)	Normal range
Alanine aminotransferase (IU/L)	Screening	22.6 ± 8.19	27.1 ± 15.30	0–41
	Final visit	26.9 ± 26.87	31.0 ± 25.18	
Albumin (g/dL)	Screening	4.5 ± 0.33	4.6 ± 0.32	3.5–5.2
	Final visit	4.4 ± 0.32	4.3 ± 1.01	
Alkaline Phosphatase (U/L)	Screening	206.2 ± 84.44	210.1 ± 97.16	53–128
	Final visit	196.1 ± 72.90	214.3 ± 75.42	
Aspartate aminotransferase (IU/L)	Screening	25.1 ± 5.95	26.4 ± 7.00)	0–40
	Final visit	27.1 ± 16.69	27.3 ± 13.79	
Blood urea nitrogen (mg/dL)	Screening	12.9 ± 3.00	13.0 ± 5.87	5.0–24
	Final visit	11.5 ± 2.75	11.4 ± 2.38	
Fasting blood sugar (mg/dL)	Screening	104.5 ± 51.03	104.1 ± 69.14	70–110
	Final visit	105.9 ± 56.70	107.2 ± 78.78	
LDL cholesterol (mg/dL)	Screening	121.6 ± 24.67	127.2 ± 42.56	Up to 140
	Final visit	123.0 ± 31.43	121.1 ± 44.51	
Potassium (mEq/L)	Screening	4.8 ± 0.41	4.7 ± 0.45	3.5–5.2
	Final visit	5.7 ± 1.76	7.3 ± 7.44	
Serum creatinine (mg %)	Screening	0.8 ± 0.12	1.2 ± 1.84	0.6–1.4
	Final visit	1.2 ± 2.07	0.8 ± 0.12	
Sodium (mEq/L)	Screening	140.8 ± 2.63	133.2 ± 30.13	136–145
	Final visit	138.5 ± 2.89	132.6 ± 21.55	
Total Bilirubin (mg/dL)	Screening	1.1 ± 0.44	1.4 ± 0.72	0.1–1.2
	Final visit	1.3 ± 0.57	1.6 ± 1.14	
Total protein (g/dL)	Screening	7.6 ± 0.53	7.6 ± 0.44	6.22–8.0
	Final visit	7.4 ± 0.46	7.4 ± 0.56	
Erythrocyte count (×10^6^ cells)	Screening	4.4 ± 0.61	4.6 ± 0.53	4.0–6.5
	Final visit	4.6 ± 0.65	4.6 ± 0.39	
Haematocrit (%)	Screening	37.9 ± 5.37	41.5 ± 3.60	40–50
	Final visit	37.0 ± 5.63	38.1 ± 8.72	
Haemoglobin (gm %)	Screening	11.9 ± 2.00	13.2 ± 1.52	11–16
	Final visit	11.7 ± 2.06	12.5 ± 2.15	
Leukocyte count (cells cu. mm-^1^)	Screening	8562.5 ± 1930.07	9233.3 ± 2369.35	4,000–11,000
	Final visit	8575.0 ± 2879.49	8157.5 ± 2413.95	
Platelet count (×10^5^ per cu. mm)	Screening	2.7 ± 0.97	2.8 ± 0.80	1.5–4.5
	Final visit	2.4 ± 0.91	2.3 ± 0.70	

Values are expressed as mean ± S.D.

**Table 6:** Change in mean vital signs from baseline to the end of study (90 days)

Vital parameter	Supplements	Baseline	Day 90 (end of the study)	Change	*p*
Systolic blood pressure (mmHg)	*B. coagulans* MTCC 5856	122.5	121.0	–1.50	0.2674
	Placebo	123.5	120.0	–3.50	0.0308
Diastolic blood pressure (mmHg)	*B. coagulans* MTCC 5856	77.0	79.5	2.50	0.1713
	Placebo	80.5	80.0	–0.50	0.7157
Pulse rate (beats per minute)	*B. coagulans* MTCC 5856	74.6	74.6	0.00	1.0000
	Placebo	76.1	74.5	–1.55	0.0841
Heart rate (beats per minute)	*B. coagulans* MTCC 5856	74.7	74.8	0.10	0.8660
	Placebo	75.8	74.8	–1.00	0.1467
Respiratory rate (breaths per minute)	*B. coagulans* MTCC 5856	21.1	21.1	0.00	1.0000
	Placebo	21.4	20.8	–0.60	0.1240
Weight (kg)	*B. coagulans* MTCC 5856	65.7	66.7	1.04	0.0589*
	Placebo	63.1	64.0	0.91	0.0785
Body mass index (kg/m^2^)	*B. coagulans* MTCC 5856	25.6	25.7	0.01	0.8956
	Placebo	25.7	25.8	0.01	0.8969

* *p*-value is estimated from paired *t*-test; *p* < 0.05 is considered as significant.

## Discussion

In our previous study, *B. coagulans* MTCC 5856 treatment exerted a significant change/decrease in the clinical symptoms of IBS such as bloating, vomiting, diarrhoea, abdominal pain and stool frequency towards end of the study (22). Consequently, this study was conducted to evaluate the effect of *B. coagulans* MTCC 5856 on the clinical symptoms of major depression with IBS. Patients who were not on any other treatment in the past 3 months for major depression with IBS were enrolled. All subjects were asked to self-administer one tablet per day (either *Bacillus coagulans* MTCC 5856 or placebo) at least 30 min before a meal, in the morning for a period of 90 days. Significant change (*p* = 0.01) in favour of the probiotic (*B. coagulans* MTCC 5856) was observed for the primary efficacy measure HAM-D, MADRS, CES-D and IBS-QOL (Table 3). This significant change in the scores of HAM-D, MADRS, CES-D and IBS-QOL is the most relevant parameter to evaluate the clinical significance of depression and IBS (33-34, 38). Additionally, statistical analysis revealed that all primary efficacy measures were statistically significant at day 60 and maintained the same up to end of the study (day 90) (Fig. 2a–d). Similarly, secondary efficacy measures also showed significant results (*p* = 0.01) in *B. coagulans* MTCC 5856 group compared to placebo group except dementia total reaction scoring (Fig. 2e–j). Overall, subjects receiving *B. coagulans* MTCC 5856 (2 × 10^9^ spores) reported a significant change/decrease in their depression clinical symptoms along with decrease in IBS towards end of the study than patients receiving placebo (Table 3). Furthermore, *B. coagulans* MTCC 5856 was also found to be very beneficial for sleeplessness and, to a lesser extent, for dementia in the current set of patients (Table 3 and Fig. 2g, h). However, these additional benefits may need to be confirmed through extensive further clinical studies on larger population of people with depression and IBS. Recent pre-clinical and clinical research reveals that the human microbiota plays a pivotal role in cognitive and affective functioning (13, 15, 20). This has led to the hypothesis that probiotic supplementation may act as an adjuvant therapy to ameliorate or prevent depression. Gut bacteria have shown to affect depression- and anxiety-like behaviour in animal models (40).

Several human studies suggested that probiotic strains have shown to improve the clinical symptoms such as cognitive performance beneficial effects on anxiety, depression and emotional arousal (13, 15, 40, 41). Moreover, these studies were conducted in healthy subjects and its clinical relevance to disease remains unclear. This is the first study to report the clinical safety and efficacy of *B. coagulans* MTCC 5856 for the management of MDD in IBS patients. However, a recent placebo-controlled trial concluded that the probiotic Bifidobacterium longum NCC3001 reduces depression but not anxiety scores and increases quality of life in patients with IBS (42). This study provides the additional evidence that probiotics may ameliorate the symptoms of depression in patients with IBS.

Myeloperoxidase (MPO) among other cellular enzymes is responsible for the production of free radicals which leads to the cellular oxidative stress (43). Oxidative stress is linked with the variety of human conditions such as rheumatoid arthritis, Alzheimer’s disease, Parkinson’s disease, cancers, cardiovascular diseases, depression and other neurodegenerative disease (43, 44). MPO has also been studied in various clinical trials as an inflammatory biomarker and used to diagnose the IBD and pro-oxidative processes in patients with depression (44, 45). By considering the clinical importance of MPO and its clinical relation with IBS and depression, we investigated the serum level of MPO in this study. In this study, myeloperoxidase demonstrated a significant decrease in *B. coagulans* MTCC 5856 (2 × 10^9^ spores) receiving patients from the baseline to the end of study (day 90) unlike the placebo group. This further validates the clinical efficacy of *B. coagulans* MTCC 5856 in patients experiencing major depression symptoms with IBS.

Despite availability of ample data, the precise mechanism of action of the probiotics in alleviating depression symptoms still remains to be confirmed. However, the basic mechanism of action in disease state (IBS) is mediated by inflammation triggered by loss of the natural eubiotic and its progression towards loss of homeostasis, that is, dysbiosis (9, 13). Further, low-grade, often chronic inflammation and/or immune activation may further increase the risk factor in mood disorders such as depression, anorexia nervosa, obsessive compulsive disorder and autism (46). However, it is also suggested that the production of certain neurotransmitters, hormones, and immune- and neuropeptides and short-chain fatty acids by the probiotics may help in alleviating depression symptoms (46). Likewise, probiotic strain *B. coagulans* MTCC 5856 has also been reported to produce short-chain fatty acids (acetic, propionic and butyric acid), antimicrobial and anti-inflammatory substances (47, 48). Thus, it may be proposed that production of short-chain fatty acids (acetic, propionic and butyric acid), antimicrobial and anti-inflammatory substances by the *B. coagulans* MTCC 5856 could be the possible mechanism of action in alleviating depression symptoms. However, further studies are warranted in order to elucidate the precise mechanism of action of probiotic strain *B. coagulans* MTCC 5856 in managing MDD in IBS patients.

In 2008, European Food Safety Authority granted the Qualified Presumption of Safety (QPS) status to *B. coagulans* (49), and the Japanese Ministry of Health and Welfare also approved *B. coagulans* for the improvement in symptoms caused by abnormalities in the intestinal flora or in dysbiosis (22, 25). Additionally, US FDA issued a ‘no questions’ letter to the GRAS notice on the use of *B. coagulans* MTCC 5856 spore preparations to be used at a maximum level of approximately 2 × 10^9^ cfu/serving in several food categories. Further to the reported safety evidences, we evaluated safety profile of *B. coagulans* MTCC 5856 at a dose of 2 × 10^9^ cfu per day in patients experiencing major depression with IBS for the 90 days of supplementation. In this trial, there were no serious AEs or significant AEs reported by the patients receiving *B. coagulans* MTCC 5856 (2 × 10^9^ spores) during the study period suggesting its safety and tolerability in this cohort of patients. There were no clinically abnormal laboratory values and changes in the vital signs between *B. coagulans* MTCC 5856 and placebo groups (*p* > 0.05). Thus, *B. coagulans* MTCC 5856 was found to be safe at a dose of 2 × 10^9^ spores (cfu) per day in patients suffering from MDD with IBS symptoms when supplemented for 90 days.

## Conclusions

This is the first investigation of *B. coagulans* MTCC 5856 as a single probiotic agent at a dose of 2 × 10^9^ spores (cfu) per day which demonstrated efficacy and safety in the patients experiencing MDD symptoms with IBS. The findings of the study indicate that the *B. coagulans* MTCC 5856 may be a new and alternative approach for the management of MDD in IBS patients. However, further prospective, larger-scale trials with extended follow-up durations are warranted in order to establish underlying mechanism as well as a detailed assessment of therapeutic effects of *B. coagulans* MTCC 5856 supplementation in managing MDD in IBS patients.
